# Traumatic Brain Injury – Modeling Neuropsychiatric Symptoms in Rodents

**DOI:** 10.3389/fneur.2013.00157

**Published:** 2013-10-07

**Authors:** Oz Malkesman, Laura B. Tucker, Jessica Ozl, Joseph T. McCabe

**Affiliations:** ^1^Department of Anatomy, Physiology and Genetics, Pre-Clinical Models for TBI and Behavioral Assessments Core, The Center for Neuroscience and Regenerative Medicine, Uniformed Services University of the Health Sciences (USUHS), Bethesda, MD, USA

**Keywords:** traumatic brain injury, animal models, depression, anxiety, irritability, aggression, suicide

## Abstract

Each year in the US, ∼1.5 million people sustain a traumatic brain injury (TBI). Victims of TBI can suffer from chronic post-TBI symptoms, such as sensory and motor deficits, cognitive impairments including problems with memory, learning, and attention, and neuropsychiatric symptoms such as depression, anxiety, irritability, aggression, and suicidal rumination. Although partially associated with the site and severity of injury, the biological mechanisms associated with many of these symptoms – and why some patients experience differing assortments of persistent maladies – are largely unknown. The use of animal models is a promising strategy for elucidation of the mechanisms of impairment and treatment, and learning, memory, sensory, and motor tests have widespread utility in rodent models of TBI and psychopharmacology. Comparatively, behavioral tests for the evaluation of neuropsychiatric symptomatology are rarely employed in animal models of TBI and, as determined in this review, the results have been inconsistent. Animal behavioral studies contribute to the understanding of the biological mechanisms by which TBI is associated with neurobehavioral symptoms and offer a powerful means for pre-clinical treatment validation. Therefore, further exploration of the utility of animal behavioral tests for the study of injury mechanisms and therapeutic strategies for the alleviation of emotional symptoms are relevant and essential.

## Introduction

Traumatic brain injury (TBI) is associated with a range of post injury complaints, and currently there are no fully effective therapies for most of these symptoms (1–4). The lack of effective therapies, the vast number of affected individuals worldwide, the loss of earning power, the cost for care, and the rehabilitative needs of patients affirms that research is essential for expanding our understanding of the pathophysiology of TBI and for developing effective treatments.

A promising strategy for the identification of the biological mechanisms underlying TBI symptomatology and facilitating the search for effective therapies is the use of animal models. Previous reviews have well described the fact that the complexity of TBI makes it difficult for a single animal model to reproduce the entire range of symptoms that occur post-TBI (5–9). Yet, animal models can mimic some of the human conditions resulting from forceful collision, non-impact acceleration, blast wave exposure, and polytraumatic events [for review, see Ref. (6)]. There are several previous, excellent reviews addressing animal models of TBI and behavior (10–13). However, the focus was primarily on cognitive and/or motor symptoms, with less attention to the neuropsychiatric symptoms frequently identified in post-TBI patients. This review focuses on the status of behavioral tests used in rodents to model depression, anxiety, irritability, aggression, and suicidal behaviors associated with TBI.

## Human TBI: The Extent of the Problem

Traumatic brain injury is a general term for a spectrum of focal and diffuse cerebral insults that result from sudden impact, changes in inertial forces, penetrating wounds, hypoxic and toxic metabolic insults, and vascular injuries of the cerebrum. Analysis of the causes and consequences of traumatic brain injuries is traditionally seen as occurring in two phases: (1) primary injury – arising from the aforementioned changes and (2) secondary injury – the biochemical and physiological brain changes caused by the primary injury, including excitotoxicity and energy failure, ischemia, cell death, edema, delayed axonal injury, and inflammation.

In 1999, the Centers for Disease Control and Prevention recognized the medical, economic, personal, and domestic consequences of TBI (14). Termed “a silent epidemic,” each year in the US, over 1.5 million people sustain a TBI (15). About 70–90% of these injuries are described as mild (16), although the percentage may be greater than this estimate with consideration that some victims do not receive treatment. Principally resulting from vehicular and home accidents or sports injuries (17), there has been continued discussion of what constitutes “mild TBI,” where complaints may include headaches, dizziness, mental “cloudiness,” a brief loss of consciousness, and post-traumatic amnesia [see Ref. (18) for further discussion of definitions]. TBI is also one of the most common injuries among US military and civilian personnel, where in the last decade an estimated 15–20% of soldiers experienced mild TBI during deployment mainly due to explosives and blast injuries (19), and almost 80% of these insults were diagnosed as mild (20).

For milder cases, the complaints usually diminish with time. Bruns and Jagoda (21), for example, cite earlier work that >58% of mild TBI victims manifest symptoms at 1 month after injury (22), but that the percentage declines to 15% by 1 year after injury (23). The latter, however, even for mild TBI cases, is a “non-trivial minority” (19). These cognitive, emotional, behavioral, and somatic problems can be debilitating and a substantial source of stress to TBI survivors and their families (24).

While there is less evidence of such long-term effects from mild injuries, neurodegenerative disorders have been associated with moderate and severe TBI (25, 26). For example, Bazarian and colleagues reviewed 75 reports regarding the long-term effects (defined as present at least 6 months post injury) from TBI (25). Dementia of the Alzheimer’s type, Parkinson’s disease, and endocrine disorders were associated with moderate and severe TBI. Post-traumatic seizures were also associated with TBI, especially if the injury was a penetrating event. For mild TBI, this group reported previous work indicating a diversity of complaints including, memory impairment, dizziness, headache, difficulties with attention, sleep disturbances, depression, anxiety symptoms, reduced stress tolerance, apathy, a lack of spontaneity, emotional excitability, and irritability (25).

Recent publications have underlined the serious, long-term effects of repeated “mild” trauma. Although much work needs to be done to define many of the specific contributors to the insidious effect from head impacts, there is evidence that repeated injuries from sport activities and possibly blast exposures, seemingly inconsequential at the time, are antecedent conditions for mood and cognitive impairments. In addition to a higher incidence of Alzheimer disease, it has been recognized that chronic traumatic encephalopathy is associated with repetitive “mild” impacts (27, 28).

Although treatment and rehabilitation of TBI has primarily focused on sensory, motor, and cognitive symptoms, co-morbid neuropsychiatric disorders (see Table 1 for a summary of the core features) that give rise to emotional and behavioral difficulties significantly impair the quality of the lives of patients and even can lead to suicide behavior (29, 30). To provide context for how behavioral testing can be employed to study post-TBI-like symptoms in pre-clinical rodent models, the clinical phenotypes are briefly reviewed. Rodent behavioral tests purportedly related to each of the aforementioned neuropsychiatric dimensions are then described, and the test’s utility in TBI research is reviewed. In general, results from the evaluation of neuropsychiatric symptoms in pre-clinical models have been limited. It is proposed that further validation is needed to relate pre-clinical models with TBI-associated neuropsychological systems.

**Table 1:** **Traumatic brain injury-related neuropsychiatric symptoms**.

Post-TBI symptoms	Prevalence (%)	Core features
Depression	10–77	Episodes of sadness, loss of pleasure, negativism, feeling of hopelessness, and suicide thoughts
Anxiety	10–70	Feeling of apprehension or dread with or without autonomic signs and symptoms. Anxiety with feelings of re-experiencing trauma, avoidant behavior, emotional numbing
Irritability	15–37	Reduced control over temper which usually results in irascible verbal or behavioral outbursts
Aggression	11–34	Destructive behaviors toward individuals or property; behaviors, attitudes, or moods that others perceive as threatening; and/or purposeful attempts to disrupt rehabilitation and discharge from hospital
Suicidality	1–17	High rates of critical indicator of suicide risk, including: hopelessness, suicide ideation, and suicide attempts

### Depression symptoms

The most common psychiatric complaint in patients with mild TBI is depression (19, 31). The reported prevalence of depression varies between studies, and ranges from 10 to 77% (32–34). Several factors contribute to the reported variability of post-TBI depression, including differences in diagnostic criteria, severity of the TBI, the time after TBI when the diagnosis is made, and diversity in patient populations (33). Rates of depression in patients with TBI are highest in the first year after the injury is sustained (35), but the risk for the appearance of depression remains elevated even decades after injury (36).

The primary symptoms of depression following TBI include episodes of sadness, negativism, loss of pleasure, feelings of hopelessness, suicidal thoughts, and sometimes psychosis (37). Victims often experience increased psychological distress and poor psychosocial status (38). In addition, depression is associated with a greater number and perceived severity of a variety of so-called post-concussive symptoms including, headache and dizziness complaints, irritability and memory problems, and sensory system-related complaints of irritability with lights and noises (39, 40).

### Anxiety symptoms

Anxiety disorders develop in 10–70% of patients with a mild TBI (19, 37, 41). As with depression, the wide range of reported prevalence is due to the nature of data collection, the severity of the brain injury, and confounding effects of demographic factors. Silver and colleagues (29) found (after adjusting for the potentially confounding effects of demographic factors, quality of life, and alcohol abuse) that individuals who experienced TBI reported a higher prevalence of panic disorder and phobic disorder compared to the control group. Fann and colleagues (39) found that 24% of outpatients with TBI had generalized anxiety disorder, while Jorge and colleagues (35) reported that 11% of the patients with TBI developed generalized anxiety disorder in addition to major depression. The core features of anxiety following TBI include feelings of apprehension or dread with or without autonomic signs and symptoms. Post-traumatic stress disorder (PTSD) is also commonly experienced by victims of TBI, and is described as anxiety with feelings of re-experiencing trauma, avoidant behavior, emotional numbing, and hypervigilance (37).

### Irritability

A prevalent post-TBI neuropsychiatric symptom is irritability (42–44). Irritability can be defined as “a feeling state characterized by reduced control over temper which usually results in irascible verbal or behavioral outbursts, although the mood may be present without observed manifestation” (45). One study found that more than 37% of the patients between the ages 18 and 65 suffered from irritability post-TBI (42), and another study indicated irritability is one of the most frequently reported symptoms by TBI patients, and that this symptom remains notable even 10 years after injury (46). Furthermore, several studies of irritability indicate it as one of the major risk factors for suicidality (47), and has a strong association with suicidal ideation and suicide attempts (48, 49). Irritability can have a significant toll on the wellbeing of the patient and their family; it often dwells in the family system and has a life-changing impact on martial relationships (50).

### Aggression

Within the Hostility Inventory developed by Buss and Durkee (51), irritability was recognized as one of the major components of an “aggression” factor. Behavioral responses to irritable states include verbal and physical aggression that contribute significantly to social and family difficulties experienced by patients with TBI (52). Broadly defined, aggression in humans is “destructive behaviors toward individuals or property; behaviors, attitudes, or moods that others perceive as threatening; and/or purposeful attempts to disrupt rehabilitation and discharge from hospital” (53, 54). There is a wide range in the current estimates of the presence of aggression in TBI patients and in a critical review of the literature, Kim and colleagues (55) concluded that these estimations are limited by a lack of a rigorous definition of the term “aggression,” as well as inconsistent methodology for identifying aggressive behaviors. In addition, family-reported aggression tends to be higher than self-reported aggression (44, 56), contributing to variability in summaries of prevalence.

Nevertheless, there is agreement that TBI increases the risk of aggressive behavior and some findings remain consistent in studies of post-TBI aggression. Aggressive behavior shows a high association with mood disorders and substance abuse (57, 58) and other co-morbid problems that were present before the injury (57). A prior-injury history of aggression was also highly correlated with post injury aggressive behaviors (52, 54). Of some fortune, aggressive behaviors displayed by brain-injured patients were most often characterized by expressions of anger and threats of violence rather than actual physical violence (58). In addition to the challenges aggressive reactivity has for the patient, family members, and caregivers, aggression may also increase the risk of co-morbid psychiatric conditions and/or suicide (59).

### Suicide

Suicidal ideation and suicide attempts post-TBI have been reported in numerous studies (60–63). One report indicates the rate of suicide in TBI patients is 2.7–4 times higher than of the general population, matched for sex and age (64). In addition, continuous military operations in Afghanistan and Iraq in the last decade have been found to be associated with increases in mild TBIs and suicides among US military personal (65). From the clinical picture, TBI patients have high rates of critical indicators of suicide risk, including hopelessness (35%), suicide ideation (23%), and suicide attempts (18%) (63).

Several studies have attempted to phenotype the association between TBI and suicide behavior, and one of the findings was the correlation between the severity of the injury and suicide behavior. A more severe injury is associated with a greater chance for suicide behavior (66–68). However, no correlation was found between suicide behavior and the injury localization (66). In addition, it was found that the risk of suicide following TBI is unceasing; even 15 years after the injury (64). However, it is important to mention that although studies frequently report suicidality is a common neuropsychological response to TBI, several studies in non-military populations did not find increased suicide risk among TBI patients (69, 70). Therefore, further exploration of the association between TBI and suicide behavior is warranted.

## Post Injury Symptoms in Animal Models of TBI

Primarily in the field of psychopharmacology, core clinical features of psychiatric diagnoses in humans have been modeled in animals. Table 2 lists the symptoms of human diagnoses and corresponding animal tests that allow for measurement of that specific symptom in laboratory animals. The data collected for this review is *limited* due to difficulty in determination of publications that did not include the test name in the title, key words, or abstract. However, this is intended as an overview of emotional systems testing in rodents after TBI.

**Table 2:** **Summary of human emotional systems and animal tests related to post-TBI symptoms in humans**.

Emotional system	Models of human symptom	Rodent test	Test parameter	TBI effect on animal performance	Number of hours/days TBI effect lasted	Reference
Depression-like symptoms	Anhedonia	Sucrose/saccharin preference	Preference for consuming sweetened fluids (sucrose/saccharin) over water	Inconclusive	7–30 days post injury	Jones et al. (79), Cope et al. (80)
	Anhedonia	Female urine-sniffing test	Preference for sniffing estrus female urine odor over water	No studies	Not applicable (N/A)	N/A
	Despair behavior	Porsolt swim test	Measurement of swimming activity during 6 min of the test	Inconclusive	7–90 days post injury	Milman et al. (81), Taylor et al. (82), Tweedie et al. (83), Jones et al. (79), Schwarzbold et al. (84), Wang et al. (85), Washington et al. (86), Shultz et al. (87), Kimbler et al. (88)
	Despair behavior	Tail suspension test	Measurement of struggling behavior during 6 min of the test, while the mouse is suspended by its tail	No effect	7 and 30 days post injury	Ando et al. (89), Vuckovic et al. (90)
Anxiety-like symptoms	Anxiety	Elevated-plus maze	Measuring the time spent in the open arm of the arena vs. the closed arms	Inconclusive	5–14 days post injury	Cutler et al. (91), Pandey et al. (92), Schwarzbold et al. (84), Shultz et al. (87), Washington et al. (86)
	Anxiety	Zero maze	Measuring the time spent in the area behind the walls vs. the open area	Inconclusive	17 days–7 weeks post injury	Ajao et al. (93), Siopi et al. (94), Tucker et al. (95)
	Anxiety	Light/dark test	Measuring the time spent in the dark area of the arena vs. the lit area	One study; less time in light chamber	∼1 week post injury	Cope et al. (80)
	Anxiety and spontaneous activity	Open field	The total distance traveled is measured (in cm); In addition, the relative amounts of time that a mouse or rat spends in the center of the open field area versus the peripheral region may also be a valid measure of anxiety	Inconclusive in rats; “hyperactivity” in mice	Increased activity −24 h–7 days post injury Decreased activity – 10–14 days Tendency to spend less time in the center	O’Connor et al. (96), Fromm et al. (97), Wagner et al. (98), Tweedie et al. (83), Jones et al. (79), Pandey et al. (92), Wakade et al. (99), Schwarzbold et al. (84), Tucker et al. (95), Kimbler et al. (88), Yu et al. (100), Budinich et al. (101), Tucker et al. (102)
Irritability-like behavior	Irritability	Resistance to capture or attempts to struggle while being restrained	The extent and duration of struggling behavior is used to measure irritability	No studies	N/A	N/A
		Responsiveness to uncomfortable stimuli	Reactivity to the stimuli	
Aggression	Aggression	Resident-intruder test	Measurement of aggressive behavior: attack bites; tail rattling; wrestling; chasing behavior; attack latency; in a 5-min of the test	One study: impaired social behavior	2 and 4 weeks post injury	Semple et al. (103)

### Depression

The two clinical hallmarks of a major depressive disorder are anhedonia and behavioral despair (71). Anhedonia is a phenomenon in which there is a loss of interest in pleasure derived from typically enjoyable experiences or activities. Behavioral despair is manifested in rodents where there is a significant decline in the animal’s effort to avoid or escape aversive situations (72). Specific behavioral tests allow these clinical hallmarks to be evaluated in laboratory animals.

#### Sucrose/saccharin preference

The sweet taste of sugar is a potent motivator both in humans and rodents (73). An often-employed, non-operant method for assessing hedonic sensitivity in rodents is the measurement of preference for consuming sweetened fluids (sucrose/saccharin) over water (74–76). Indeed, rodents will not only consume a freely available sweet solution, they will perform a variety of tasks to obtain rewarding solutions: press levers, run down an alley, etc. In general, rodents will work harder as the concentration of the sweet solute is increased (73).

Rodents normally exhibit very high preferences for the sweeter solution, but this preference diminishes after exposure to chronic mild stress or when they exhibit other depression-like symptoms. Willner and colleagues (74) proposed that reduced consumption of sweet solutions by rats after chronic mild stress is a measure of anhedonia. Interestingly, studies have found that different kinds of stressors had distinctive effects on saccharin consumption in rats. Physical (foot shocks) stress resulted in rats having a reduced preference for saccharin solution, while after emotional stress (presence in the adjacent compartment during foot shock treatment of their cage mate), animals displayed a slight increase in sweet solution preference (77).

A variety of saccharin/sucrose preference tests have been described, but the tasks are fundamentally the same. The animal is presented with two bottles; one bottle contains tap water and the other a sweet solution (saccharin or sucrose; the concentration is determined according to the strain of the animals and the lab protocol, and varies from 0.004 to 0.35%). The animals have the opportunity to drink as much of the solution or water as they want during a significant amount of time (at least overnight). The bottles are weighed before they have been introduced to the animals and again at the end of the experiment, and a preference ratio is calculated (consumed sweetened solution)/(consumed water + consumed sweetened solution). The preference for sweet solution over water exhibited by the rodent is used as a measure for sensitivity to reward (76). Although the sweet solution preference test is widely used, it has limitations. Forbes and colleagues (78) showed that reduced consumption of sweet solution in stressed rats might result solely from diminished body weight, rather than stress *per se*, and concluded that sucrose consumption cannot be used as a valid test for hedonic responsiveness. In addition, differences in this test may result from alterations in the gustatory system and appetite by gene manipulation and/or pharmacological treatment, and may not truly indicate a change in the reward-seeking state.

Jones and colleagues (79) have explored the effects of TBI on depressive-like symptoms in rats (lateral fluid-percussion injury model; ∼3.5 atmosphere pressure pulse directed to the right sensorimotor cortex), and found that there were no significant differences between injured rats and sham controls in the sucrose preference test, 1, 3, and 6 months after the primary injury (79). On the other hand, Cope and colleagues found that TBI rats [controlled cortical impact (CCI) to the frontal cortex; 5 mm impactor tip, 3 mm depth, 2.25 m/s, impact time 500 ms] showed a significantly lower preference for saccharin compared to the control sham rats 8 days following injury (80). The number of studies using the sweet solution preference test in rodent models of TBI is, unfortunately, very limited, and therefore it is premature to assert the utility of this measure for TBI rodent models.

#### Female urine-sniffing test

The female urine-sniffing test (FUST) is a non-operant method that measures hedonic behavior using a sexual incentive (104). The FUST procedure combines two previous areas of investigation: the olfactory habituation/dishabituation test (72, 105, 106) and the use of estrus urine or bedding as a sexual incentive (107, 108). Studies have shown that estrus female rodent urine extracts elicit specific behavioral displays analogous to those observed in the presence of reward incentive stimuli: increased number of approaches, longer time spent with the stimulus, and shortened approach time to the stimulus [for review, see Ref. (109)].

One hour prior to the test, rodents are habituated to a sterile cotton-tipped applicator inserted into their home cages. After 1 h, the sterile cotton-tipped applicator is removed and each rodent, separately, is transferred to a dimly lit room (∼3 lux lighting). The FUST has three phases: (1) water – one exposure (3 min) to a cotton tip dipped in sterile water, during which sniffing duration is measured; (2) habituation – the rodent cage is transferred from the experiment room to an habituation area for an interval of 45 min during which no cotton tip is presented; and (3) urine – the animal is transferred back to the experiment room for one exposure (3 min) to a cotton tip applicator infused with fresh urine collected from females in estrus (of the same strain), and sniffing duration is measured.

The FUST has been validated in several rodent strains (C57BL/6J mice, 129S1/SVImJ mice, and Wistar-Kyoto rats). It has been employed with several pre-clinical experimental paradigms including, learned helplessness (LH) (110), to evaluate the effects of chronic treatment with a selective serotonin-reuptake inhibitor (SSRI) with mice that experienced the LH (104), after single electric shock stress manipulation (104), and with a mouse strain (GluR6KO) that exhibits manic-like symptoms (111). GluR6KO males spent a significantly longer duration sniffing female urine compared to wild-type control mice (104). The FUST is suitable and well validated for measuring hedonic-like behavior in male rodents. However, due to the novelty of the test and the short time of its existence, there are no available data for the effects of TBI in mice or rats.

#### Porsolt forced swim test

The forced swim test was developed by Porsolt and colleagues (112) and has become widely used for the study of rodent models of depression or “behavioral despair” (113), response to stress (114), and as a tool for screening antidepressant drugs (112, 115, 116). In the forced swim test, the rodent is placed in a cylinder filled with water with no ability to escape. Typically, animals initially paddle vigorously, but then become relatively immobile (defined as a lack of activity except movements needed to keep the nose above water), and finally may adopt a characteristic vertical floating response. Abel (116) has demonstrated that the vertical immobility response in the forced swim test has a sudden ontogenetic onset, beginning at 21 days of age and quickly stabilizing at 26 days of age.

The procedure may vary for mice and rats. Mice are only immersed once into the cylinder of water for session durations between 4 and 20 min [see Ref. (72) but also, see Ref. (117)], in which a pre-exposure period of 2–5 min is conducted before the swimming activity is measured. Rats usually are immersed twice in the water cylinder where initially the rats are immersed for 10 min, in which no measurements are taken. Twenty-four hours later, rats are re-immersed for 6 min and swimming activity is evaluated. However, in some variations of the test, rats are only immersed once and immobility time is recorded during this single session (118–120).

Several studies have found that prepubertal and adult rodents exhibiting depression-like symptoms on several behavioral tests, such as the saccharin preference, also exhibited longer immobility duration in the Porsolt swim test (114, 121–123). This increased immobility duration can be altered by acute and chronic antidepressant treatment (121, 124, 125), making the Porsolt forced swim test appropriate for the study of depression-like symptoms in rodents.

Compared to the sweet solution preference tests and the FUST, the swim test has been frequently employed to evaluate rodent performance after TBI. Milman and colleagues (81) found that injured mice showed significantly greater durations of immobility compared to sham controls 7 and 90 days after TBI induced by a weight-drop device (metal weight of 30 g dropped vertically from 80-cm height). Taylor and colleagues (82) found that after 8 weeks, rats with TBI induced by CCI (left parietal cortex; 5 mm diameter impactor tip; 28 psi pressure; 2.75 m/s for 250 ms) exhibited significantly less swimming behavior compared to the sham controls; also suggesting depression-like symptoms in brain-injured rodents. In addition, clear differences between Imprint Control Region (ICR)-strain mice with a mild TBI (induced by a 30 or 50 g metal weight dropped from a height of 80 cm) and their sham controls were found in a study conducted by Tweedie and colleagues (83), where injured animals spent a longer time immobile when tested 3 days after the injury. Lastly, Washington found CCI (3.5 mm tip, 5.25 m/s speed, 0.1 s dwell time, 1.5–2.5 mm depth) resulted in a significant increase in immobility time 21 days after injury (86). It is noteworthy to mention that several studies have not found any significant differences in the forced swim test after TBI. Jones and colleagues (79) found no difference in swimming activity between sham controls and rats receiving a TBI to the right sensorimotor cortex (lateral fluid-percussion injury; ∼3.5 atmosphere pressure pulse) 6 months post-TBI. Schwarzbold and colleagues (84), did not find a significant difference between TBI mice and their sham controls, 10 days after the injury induction (weight-drop TBI device, weights varies between 10 and 15 g, with a diameter of 3 mm, and dropped from 120 cm height on the left parietal region). Wang and co-investigators examined the effect of moderate to severe TBI (−2.0 mm AP and 2.0 mm ML relative to the bregma suture) of C57BL/6 mice, produced by CCI (2.0 mm diameter tip; 1.5 m/s velocity; 1.25 mm depth; 155 ms contact time), and found no difference between brain-injured mice and their sham controls in the forced swim test, 4 weeks post injury (85). Shultz found that only after five injuries by fluid percussion, mice exhibited an increase in immobility score 8 weeks post injury (87). Kimbler observed that, surprisingly, CCI (4.5 m/s, 20 ms dwell time, 1 mm depth, 3 mm diameter impactor tip) lengthened the latency to immobility 72 h after injury (88).

The causes for different outcomes from TBI may be related to an oft-cited problem in behavioral research in general; laboratories employ different experimental conditions. Test procedures vary across studies, different strains of animals were used, the time after injury when rodents were evaluated, and the procedure for inflicting TBI and the location of the injury differs across laboratories. Thus, further studies are needed to assess the impact of TBI on performance on the forced swim test.

#### Tail suspension test

The tail suspension test has been employed as a measure of depression-like behavior (126). As in the case of the Porsolt swim test, this test is based on the assumption that the emotional state of an animal will influence their efforts to escape an aversive situation, and that the internal states relates to the duration of the escape response (72). In this test, the aversive situation accrues while the mouse is being suspended by the tail, usually for 6 min, while the body suspends in the air facing downward. The duration of struggling to face upward and reach for a solid ground is recorded and compared between mice/strains. Usually, a naïve mouse will struggle for several minutes, but eventually stops moving and remains immobile for the remaining time of the testing session (72).

Few TBI studies have employed the tail suspension test, and further study is needed to determine the effect of TBI on the rodent’s behavior in this test. Ando and colleagues (89) found that CCI (2 mm tip; 4.5 m/s velocity; 2 mm depth; 80 ms contact time) followed by laser treatment reduced immobility time with the tail suspension test 4 weeks after the injury, but the study did not include a sham-treated or naïve control group for comparison. Brain injury by exposure to 1-methyl-4-phenyl-1,2,3,6-tetrahydropyridine (MPTP) was found to have no impact upon the performance on the tail suspension test 7 and 30 days post injury compared to the control group (90).

### Anxiety

Several established procedures for measuring emotional reactivity and anxiety-like symptoms in rodents are based on approach-avoidance situations where anxiety alters responses to a conflict inherent in the test setting (72). Here, we will review the most well-known tests.

#### The elevated-plus maze

The elevated-plus maze (EPM) is a widely employed measure in anxiety research (127) and an accepted procedure for drug discovery in pharmaceutical companies (128). Rodents have a natural tendency to explore novel environments, but brightly lit, open, elevated areas are perceived by the animal as aversive. The EPM measures the naturalistic conflict between these two features (129).

The maze is usually constructed of Plexiglas in a plus-shaped formation with two dark, enclosed arms and two open well-lit arms, elevated 50–100 cm above a table surface or floor. The dimensions of the arms are usually 30 cm × 5 cm with a 5 cm × 5 cm center area, and the walls of the closed arms are ∼40 cm high. A single mouse is placed in the center of the maze for 5 min, and usually tracked with a video camera. The time spent in the three different areas of the maze (closed arms, open arms, and center) and the frequency of visits to these different zones is scored manually or by the use of automated software. Compared to control animals, rodents exhibiting anxiety-like behaviors spend more time in the closed arms of the apparatus than in the open arms. The validity of the test is supported by studies that show performance is sensitive to anxiolytic and anxiogenic compounds: anxiolytic drugs (e.g., benzodiazepines) typically increase the percentage of time spent in the open arms relative to time spent in the open and closed arms together, anxiogenic drugs decrease the amount of time the animals spend in the open arms (127, 130).

Schwarzbold and colleagues (84) found that mice 11 days after a TBI induced by a 10-g weight dropped on the left parietal region (from a height of 120 cm) exhibited a significant decrease in entries into the open arms, and spent less time in the open arms (anxiety-like behavior) compared to sham controls and to mice with TBI induced by 12.5 and 15 g weight dropped on the same brain region, suggesting that a mild TBI, but not more severe injuries, may result in an elevated anxiety response. On the other hand, Pandey and colleagues (92) found that 14 days after a mild TBI (400 g weight, 10 mm diameter, dropped from a height of 1 m on the exposed skull, mid-distance between lambda and bregma sutures), rats exhibited a significant increase in both percentage of open-to-total arm entries and percentage of total time spent in the open arms when compared with rats that underwent sham surgery. Although both of the latter studies have used different species, each claimed to induce “mild” TBI by using the weight-drop procedure. However, the parameters used in these studies are substantially different, and hence may underlie the differences in behavioral results. An estimate of kinetic energy of the weight drop in the Pandey study (92) indicates it may have been three-times greater than what was employed in the Schwarzbold experiment (84).

Cutler and colleagues (91) found no difference in EPM performance between vehicle-repeated injections treated injured rats induced by CCI (5 mm diameter; 1.7 psi; 50 ms; 2.25 m/s) and vehicle-repeated injections treated sham controls 5–6 days after injury. But, rats induced by CCI and vehicle treated in silastic capsule implantations spent more time in the open arms compared to vehicle treated in silastic capsule implantations sham controls. In addition, Shultz and colleagues (87) used the EPM and found that repeated fluid-percussion injury (FPI) resulted in a decrease in time spent in the open arms at 24-h and 8 week timepoints post-TBI (87). Washington and colleagues (86) measured the duration of mouse exploration in the open arms 21 days after TBI, and found that mild, moderate, and severe TBI animals (induced by CCI) spent a significantly greater amount of time in the open region – hence, showing reduction in anxiety-like behavior and increase in risk-taking-like behavior (86), compared to their controls, with no effect related to the extent of the injury. Thus, the effect of TBI on anxiety-like behavior in the EPM in mice and rats is unclear and further studies are needed.

#### Zero maze

Considered a “modification” of the EPM, the zero maze has been used to evaluate anxiety-related behaviors in rodents (131), and in the last year has seen utility in rodent model studies of TBI (93–95). Similar to the EPM, the zero maze is brightly lit, with alternation between dark areas surrounded by walls, and open areas along an elevated circular runway. As opposed to the EPM, the zero maze has no central start box, in which mice often remain for significant periods of time; hence avoiding the ambiguity in scoring arm entries and reducing variability of the data (72).

Ajao and colleagues report that when 17 day-old rats sustained CCI (3-mm diameter impactor, 1.5 mm depth, 200 ms duration at 6 m/s velocity), they exhibited an increase in time spent in the dark (walled) regions of the maze compared to sham-treated animals 60 days after injury (93). Siopi and colleagues performed weight-drop injuries (50 g weight, dropped from 36 cm height) on mice and compared their performance to naïve-treated animals (94). Injured mice exhibited no change in the percentage of time or the number of entries into the open portion of the zero maze, but they exhibited a greater number of U-turns, 7 weeks post injury. Tucker and coworkers exposed male mice to a single 900 mV, 10 ms wave generated from a high intensity focused ultrasound device (95). Ultrasound exposed mice exhibited reduced time in the open regions of the zero maze 4 days post injury, compared to sham-treated animals. Overall, it seems that TBI has an anxiolytic-like effect on rodent behavior in the zero maze test, but further study is needed.

#### Open field test

The open field test, originally developed by Hall (132), has become one of the most frequently employed tests for measuring spontaneous activity (72). Investigators have traditionally employed this test as a measure of “emotionality” (132–134). In the open field procedure, the animal is initially placed in the center of the apparatus (usually 40 cm × 40 cm × 30 cm; W × L × H box) and the following behaviors are recorded for 2–20 min: horizontal locomotion, frequency of rearing, grooming, freezing, number of fecal boli deposited, and the proportion of time spent in the center of the arena (72).

In addition to being the most widely used assessment of general activity, the open field test was one of the earliest tools that was applied to evaluate the incidence of freezing and defecation as measures of anxiety-like behaviors (135, 136). The relative amount of time that a mouse or rat spends in the center of the open field area versus the peripheral region is likewise considered a measure of anxiety; rodents are exploratory but will generally stay near the walls of the open field [for review, see Ref. (72, 137)]. There are two naturalistic factors that trigger anxiety-like behaviors in the open field: agoraphobia (the arena is very large relative to the animal’s natural environment) and individual testing (the animal is separated from its social group). These two factors trigger anxiety-like behavior in rodents that live in social groups and in small tunnels (138). Pharmaceutical agents such as benzodiazepines, serotoninergic ligands, opiates, and dopaminergic agents all decrease anxiety-like behaviors of rodents in the open field, suggesting that this test is appropriate for studying anxiety in mice and rats (139–142).

Many studies have explored the effects of TBI on motor activity and anxiety-like behaviors of rodents in the open field. Rats with a TBI in the right parietal cortex induced by CCI (6 mm impact tip diameter; velocity 4 m/s) were no different than sham controls 13 days after injury on measures of activity and exploratory behavior in the open field (98). Another study (79) employed the FPI model and also found no significant differences between injured rats (FPI −4 mm lateral and 4 mm posterior to bregma; 3.2–3.5 atmospheres of pressure pulse over 21–23 ms) and their controls. These animals were evaluated for the total distance traveled in the apparatus, 1, 3, and 6 months after injury. However, injured rats spent less time exploring the center of the open field compared to sham controls, suggesting some difference in level of anxiety as a function of FPI. Fromm and colleagues (97) showed that TBI rats, induced by a 450-g weight dropped from a 2-m height on a 10-mm diameter disk (placed on the exposed skull, between the lambda and bregma sutures), exhibited significantly less spontaneous activity in the open field 7 days post injury, compared to pre-injury results. Using the same injury method and parameters, measures found injured rats performed significantly poorer in activity measurements as well as anxiety-like behavior measurements. TBI animals were more stationary; traveled less distance in the arena; less explorative; explored only the corners of the apparatus. Another study, using similar weight-drop parameters, found persistent decreased open field activity for up to 4 weeks after injury (96). As opposed to the studies mentioned above, Pandey and colleagues (92) found that injured rats exhibited increases in several activity parameters in the open field model, 14 days after injury induction, compared to their sham controlled rats.

Activity level changes in mice follow a more consistent pattern. Wakade and colleagues (99) found that CD-1 mice, 7 days following a moderate TBI induced by CCI (3.5 mm craniotomy midway between the bregma and lambda), exhibited hyperactivity in the open field test, compared to their sham controls. Kimbler and colleagues observed that CCI-injured mice exhibited an increase in distance traveled compared to sham-operated animals 3 days after injury (88). Yu and colleagues examined the effects of brain injury 10 days after post-CCI (3 mm tip, 2 mm depth, 5 m/s); the injury resulted in mice that were hyperactive in terms of distance traveled, but the animals spent less time in the center (100). Budinich and colleagues reported that CCI (3 mm impactor, 1.0 mm depth of impact, 5 m/s, dwell time of 0.1 s) caused mice to exhibit increased mobility on 1, 7, and 14 days after injury, but that they spent less time in the center of the open field (101). Similar instances of “hyperactivity” have been observed in our laboratory for several weeks post-TBI: Tucker and colleagues (102) studied mice that sustained either mild (impact depth 1.0 mm) or severe (depth 2.0 mm) CCI (3 mm impactor tip, velocity 5 m/s). Schwarzbold and colleagues found similar results: 10 days after TBI induction, injured mice (of the 15-g weight group) exhibited higher locomotor activity compared to mice from other control groups (84). Animals that sustained severe CCI were hyperactive with less time spent in the center. Following mechanical stimulation by HIFU, however, animals were hypoactive in the open field (95) for at least 1 week post-TBI. Likewise, Tweedie and colleagues found no differences between sham-treated and injured mice in the percentage changes in distance traveled by mice 3 days after TBI (83).

The results from rat studies, regarding activity measurements in the open field test after TBI induction, are inconclusive. It is important to note that different laboratories do not only use different models of TBI, but also use different strains of rats (e.g., CD-1, Wistar, Sprague-Dawley, etc.). Therefore, additional research of the effect of TBI on activity, as well as on anxiety-like behavior in the open field test is essential. For studies with TBI in mice, there is greater consistency in findings where the general finding is an increase in activity.

#### Light/dark test

Analogous to the elevated-plus maze, the light/dark test is based on the conflict between the tendency of mice to explore a novel environment versus the aversive properties of a brightly lit open field (143). The typical dimensions of the light/dark test apparatus for mouse use are 46 cm × 27 cm × 30 cm (L × W × H) and it is divided into two parts: an opaque, covered chamber, considered by a rodent to be the “safe” compartment (approximately one-third of the apparatus), and a larger, illuminated “aversive” compartment, which is a transparent, uncovered arena that is illuminated by an overhead lamp. Rodents that exhibit anxiety-like behavior will spend significantly more time in the dark, “safe” compartment compared to their controls. Crawley and Goodwin (143) showed that anxiolytic drugs (benzodiazepines) facilitated exploratory behavior; mice spent more time in the illuminated open area as a result of the treatment. Indeed, multiple subsequent studies have shown that anxiolytic drugs increase locomotion and time spent in the light “aversive” compartment, while anxiogenic compounds decrease locomotion and time spent in this compartment [for review, see Ref. (144)]. Few studies appear to have investigated the effects of TBI on the behavior of rodents in the light/dark test. Cope and colleagues report that TBI rats (induced by CCI to the medial frontal cortex) spent less time in the lighted arena and made fewer crossings between the different zones 8 days after injury (80), exhibiting anxiety-like behavior.

### Irritability

“Irritability” has been recognized as a state that can be evaluated in rodents. It has been described as a condition where the animal exhibits “wild” and/or restlessness in response to a perceptible stimulus (145), and as an extreme reaction to relatively mild stimuli (146). However, as opposed to aggression and impulsivity, irritability – in humans – is not just behaviorally defined, but involves subjective state changes (147); therefore, the modeling of irritability in rodents is conceptually complicated. A variety of drugs such as parachlorophenylalanine (148) and trimethyltin (149), and imidazole (150) can provoke this state. Likewise, psychoactive drug withdrawal, especially for opiate drugs, reportedly most robustly elicits this state (151, 152).

The two most commonly used tests for evaluation are resistance to capture or attempts to struggle while being restrained (153), and responsiveness to uncomfortable stimuli. Tests also include assessing rodent struggling behavior in response to human handling. As a response to moderate restraint applied by the handler, a mouse will either exhibit a passive or irritated response. The extent and duration of struggling behavior are used as metrics (153). Tests of responsiveness to a uniformly uncomfortable situation have also been employed. An uncomfortable stimulus is given (e.g., a white bottle brush moving against the animal or a puff of air blown sharply through a straw onto the back of the animal’s neck) and the animal’s response is quantified. Animals that exhibit enhanced reactivity to the stimuli are considered to display irritable behavior (146). Irritability as a post-TBI symptom is undoubtedly important clinically and testing in animal models may shed light on this complex symptom. However, a review of the literature found no studies that have examined this post-TBI.

### Aggression

Beginning in the juvenile phase of development, rodents establish dominance hierarchies, and engage in fighting behavior (154). Indeed, rodent aggression behavior has been described in detail in many laboratories (155). Experts suggest laboratory studies with rodents mirror at least some aspects of aggression in humans (156). Several behavioral tests assess aggression in rodents, including the tube test (157) and the standard opponent method (158). However, the most widely employed assessment is the resident-intruder test (159).

#### Resident-intruder test

In the resident-intruder test, an “intruder,” usually a naïve, weight- and age-matched mouse (weight and age are crucial components affecting aggression behavior in rodents), is placed into “the home cage,” that the experimental mouse has been housed in for an extended period of time: the intruder elicits territorial attacks from the male resident in the home cage (72). Usually, a 5-min resident-intruder session is conducted [although test sessions vary from 2 to 30 min (160)] and videotaped recordings are used for subsequent scoring by the investigator. The investigator scores the frequency and the length of the following behaviors: attack bites – biting of the intruder mice; tail rattling – rapid lateral quivering of the tail, just before or after attacking; wrestling – vigorous shoving and sparring when both animals take on an upright posture, usually performed by both animals simultaneously; chasing behavior – rapid pursuit of the intruder by the test male, with or without physical contact; and attack latency – latency time to the first attack (in seconds) from the introduction of the intruder mouse.

Numerous studies have found that acute (but not chronic) treatment with antidepressants has been able to reduce aggressive behavior in the resident-intruder test in rats, including: SSRIs, serotonin-norepinephrine reuptake inhibitors, monoamine oxidase inhibitors (MAOIs), tricyclic antidepressants (TCAs) and atypical antidepressants such as mainserin and iprindole (161). Mouse aggressive behavior, on the other hand, has been found to be sensitive to anxiolytic drugs rather than antidepressants in the resident-intruder test (162). Lumley et al. (162) proposed that this difference in response between mice and rats is due to the functional differences in aggressive behavior between these species: while mice violently defend their territory, rats live in social colonies where excessive agonistic behavior is harmful to the social group.

Semple and colleagues (103) evaluated social behavior, including aggressive behavior, in mice subjected to TBI at post-natal day (pd) 21. The resident-intruder test was conducted twice – once in the adolescence phase (pd 35–42) and during early adulthood (pd 60–70). The researchers found that despite normal olfactory function and normal social behavior during adolescence, injured mice exhibited impaired social investigation in the resident-intruder test by adulthood; injured mice displayed reduced anogenital sniffing and following compared to their controls. In addition, by adulthood, injured mice showed more frequent dominance in the tube task compared to their sham controls, suggesting aggressive tendencies in the TBI mice. No studies have been conducted on the effect of TBI on aggression behavior in rats. However, lesion studies have been a classic approach for delineating systems related to aggression and integration with this field is warranted (163, 164).

### Suicide

Suicide is a complex human behavior and is an aftereffect in many cases of TBI. Unfortunately, there are no existing animal models for suicide *per se*. Studies have tried to identify personality traits that are risk factors for suicidal behavior (165, 166), and the four major risk factors that have been found to be common for most of the studies were aggression, impulsivity, irritability, and hopelessness/helplessness [for a thorough review of these risk factors and their animal models, see Ref. (161)]. As reviewed earlier, all of these behaviors are amenable to modeling in rodents, providing an opportunity to determine neurobiological mechanisms relating brain injury and suicidality.

## Summary

The simulation of TBI-relevant neuropsychiatric symptoms in pre-clinical rodent models is a critical step for further understanding basic molecular and structural relationships and for the development of therapeutic approaches. As noted earlier, some symptom profiles in the realm of motor and cognitive function have received wide employment in pre-clinical TBI research. For example, this literature review found popular and suitable motor tests include the beam walk test, rotarod, and neurological assessment scales. Likewise, the Morris water swim test and open field test are conventionally employed for cognitive assessment. Neuropsychiatric impairments have received less attention in animal models of TBI and are recognized as a challenge. Table 2 summarizes the status of emotional systems testing in rats and mice after TBI. The literature suggests “hyperactivity” is seen in the open field test in mice after TBI, and one study found anxiety responses in the Light/Dark Test. One study has employed the Resident-Intruder Test as a measure of aggression and social behavior. However, few tests were found that employed other tests of emotional systems that have received quite wide use in the psychopharmacology field.

Rodent models cannot replicate “higher” cognitive-emotional reactions and expectations of individuals and their social milieu, and how the patient must learn to cope with the knowledge of personal mental and/or physical impairment(s) and potential limitations in adjusting to life’s many challenges. Animal models, then, have limitations in ability to fully mimic the human condition. Likewise, “There is no way for a human investigator to know whether a mouse is feeling afraid, anxious, or depressed… . What we can do is observe the behavioral and physiological responses that a mouse makes to stimuli and events” (72). In spite of limitations, neuropsychiatric symptomatology from TBI related to depression, anxiety, irritability, and aggression are major aspects of human suffering, and require continued and concerted efforts related to etiology and therapeutics.

## Conflict of Interest Statement

The authors declare that the research was conducted in the absence of any commercial or financial relationships that could be construed as a potential conflict of interest.
